# Supplementary material for: Thawing permafrost poses environmental threat to thousands of sites with legacy industrial contamination
Source: Nat Commun. 2023 Mar 28;14:1721. doi: 10.1038/s41467-023-37276-4 (PMC10050325; doi:10.1038/s41467-023-37276-4)
Supplement: Supplementary file 1 — Supplementary Information [file 41467_2023_37276_MOESM1_ESM.pdf]

Supplementary Information for:

Thawing permafrost poses environmental threat to thousands of sites with legacy industrial contamination

Moritz Langer*[1,2], Thomas Schneider von Deimling[1,3], Sebastian Westermann[4,5], Rebecca Rolph[1,3], Ralph Rutte[6], Sofia Antonova[1], Volker Rachold[7], Michael Schultz[8], Alexander Oehme[1,3], Guido Grosse[1,9]

1 Permafrost Research Section, Alfred Wegener Institute Helmholtz Centre for Polar and Marine Research, Telegrafenberg A45, 14473 Potsdam, Germany
2 Department of Earth Sciences, Vrije Universiteit Amsterdam, Amsterdam, the Netherlands
3 Geography Department, Humboldt-Universität zu Berlin, Unter den Linden 6, 10099 Berlin, Germany
4 Department of Geosciences, University of Oslo, Sem Sælands vei 1, 0316 Oslo, Norway
5 Centre for Biogeochemistry in the Anthropocene, University of Oslo, Norway
6 Freelancer, Nonnenwaldstr. 10, 65817 Eppstein, Germany
7 German Arctic Office, Alfred Wegener Institute Helmholtz Centre for Polar and Marine Research, Telegrafenberg A45, 14473 Potsdam, Germany
8 GIScience, Heidelberg University, Im Neuenheimer Feld 348, 69120 Heidelberg, Germany
9 Institute of Geosciences, University of Potsdam, Karl-Liebknecht-Str. 24-25, 14476 Potsdam, Germany

Corresponding author: Moritz Langer

Email: moritz.langer@awi.de

Supplementary Tables

Supplementary Table 1: Overview of different types of toxic industrial substances stored, spilled, or lost in or on top of permafrost based on a sample of the literature.

Sector	Activity group	Example of pollutants / damages	References
Energy	Resource exploration and transportation	Drilling fluids (surfactants, detergents, large quantities of highly concentrated saline solutions primarily potassium chloride), fuel	1-6
Industrial Production and Product Use (IPPU)	Mining Military	fuel, radioactive waste, acid mine drainage (Al, Mn, Cu, Hg), flotation chemicals, mining leachate	7-18
Waste	Industrial landfills	Heavy metal contamination (e.g. Cadmium, Mercury, and Lead), organic waste, synthetic materials	19-24
Agriculture, Forestry and Other Land Uses (AFOLU)	Pollution	Deforestation, fertilizers, pesticide, urbanization, fuels	25
Other	Accidental	Hydrocarbon (oil, diesel, etc) spills, nuclear accidents and non industrial wastes	26-30

Supplementary Figures

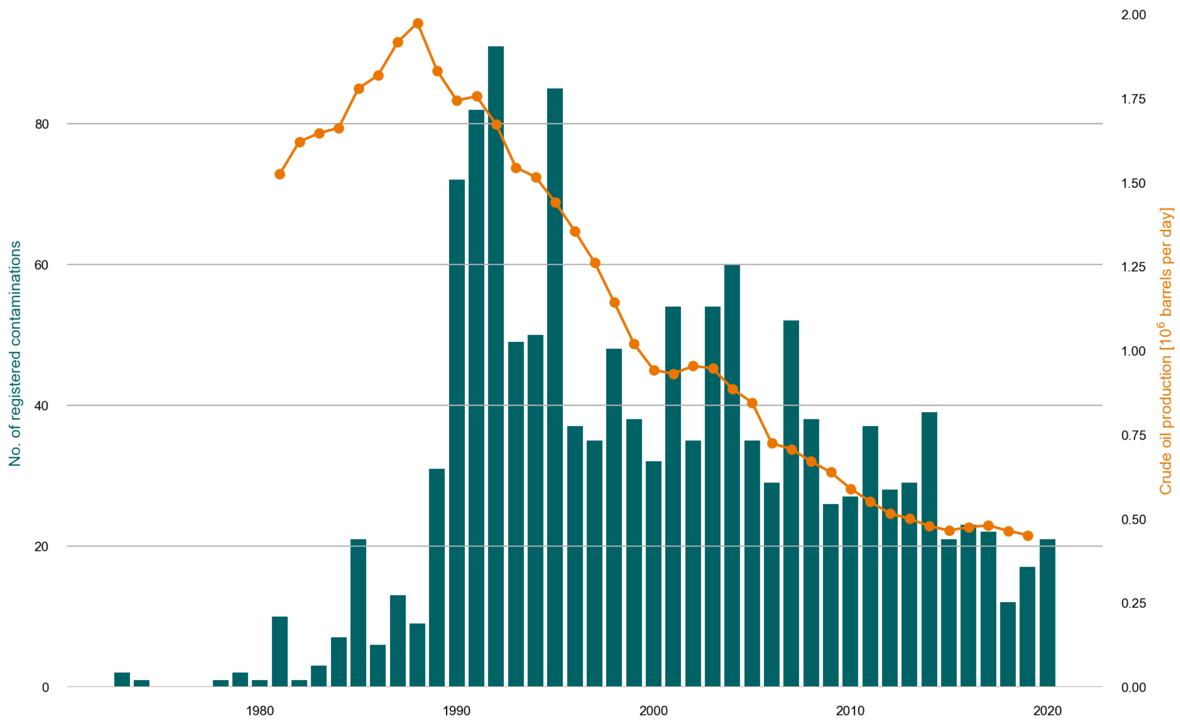

Supplementary Fig. 1: Number of contaminations in the permafrost dominated region of Alaska according to the first date of registration within the CSP together with quantities of crude oil production on the North Slope as provided by the U.S. Energy Information Administration (EIA) (31).

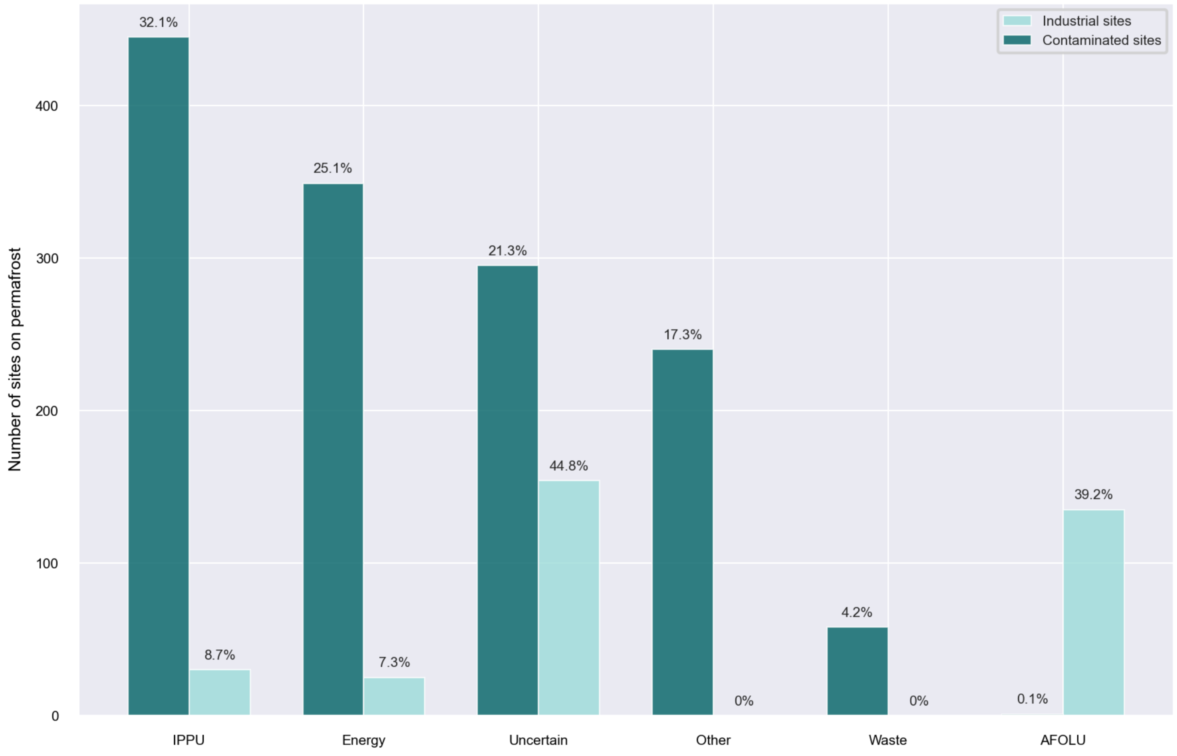

Supplementary Fig. 2: Occurrence of industrial sites and contaminated sites in permafrost dominated region of Alaska according to industrial sectors defined by the semantic classification undertaken (see Supplementary Fig. 7). Numbers on the bars indicate relative occurrence. More than 50% of the existing contaminated sites are attributed to industry within the sectors Industrial Processes and Product Use (IPPU) and Energy which together represent less than 20% of the existing industrial sites. Agriculture, forestry, and other land uses (AFOLU) account for the largest number of known industrial sites in Alaska.

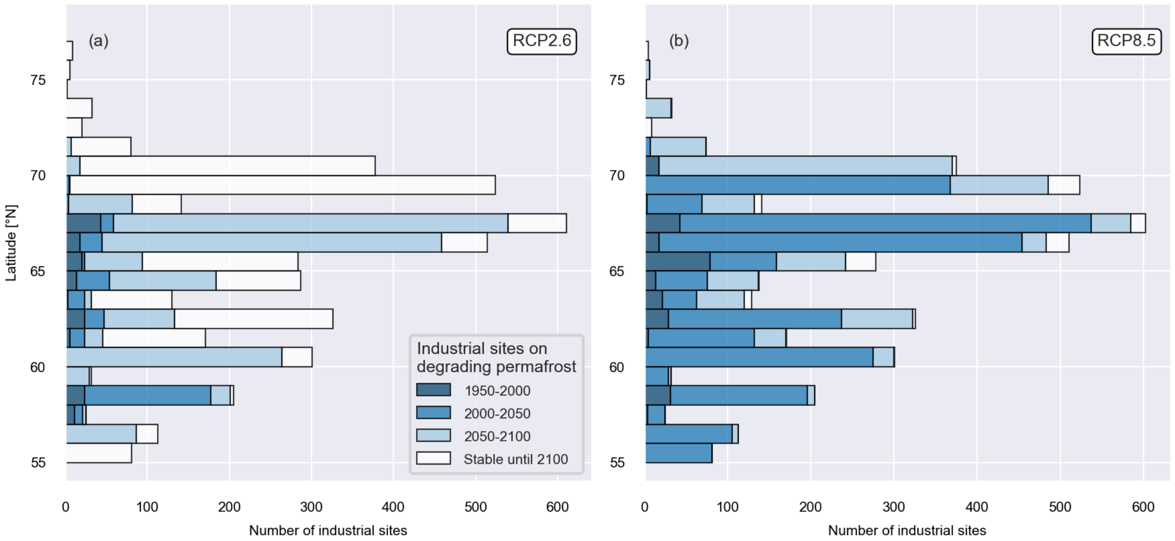

Supplementary Fig. 3: The latitudinal distributions of industrial sites that experience permafrost degradation in different time frames illustrated for the (a) “best case” (CCSM4, RCP2.6) and (b) “worst case” scenario (HADGEM2-ES, RCP8.5).

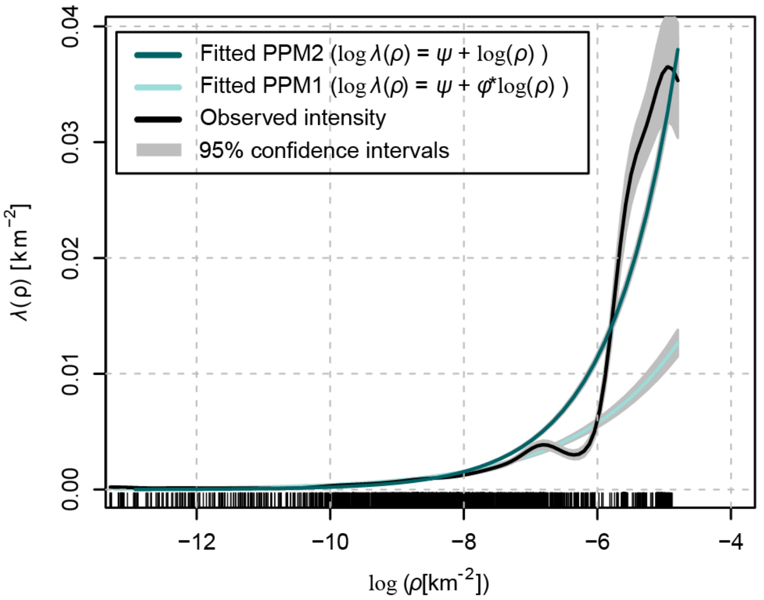

Supplementary Fig. 4: The observed and modeled point intensities of contaminated sites λ with the point density of industrial sites ρ for the permafrost dominated regions of Alaska and Canada. Two basic inhomogeneous poisson point models (PPM1 and PPM2) are applied where the covariate ρ is the density of industrial sites, ψ and φ are unknown scale parameters derived by model fitting, and $\lambda(\rho)$ is the expected conditional intensity of contaminated sites. The shaded areas around the curves relate to the 95% confidence intervals of the fitted parameters. The models do not exactly reproduce the observed spatial relationship, but mark the upper and lower limits given by the large variations at higher industrial site densities.

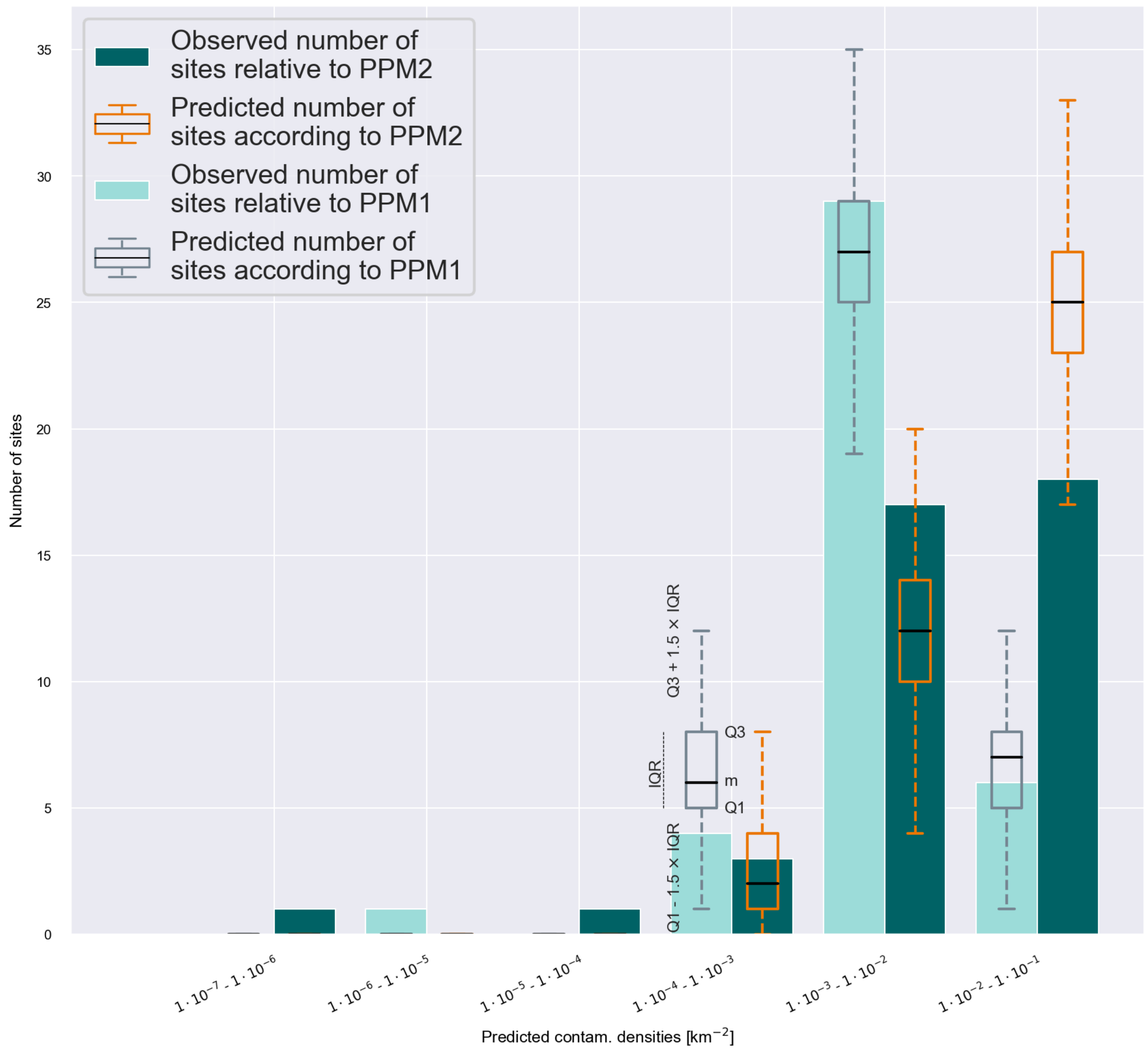

Supplementary Fig. 5: The number of identified contaminated sites in Russia that are located within the predicted intensity classes of the two point process models. To verify that the observed number of contaminated sites matches the expected number according to the models, multiple (N=1000) random samples (n=44) were drawn from the entire model distribution for Russia (shown as a boxplot). The models reproduce the observed distribution well, mostly within the interquartile ranges (boxes) and always within 1.5 times the interquartile ranges (whiskers).

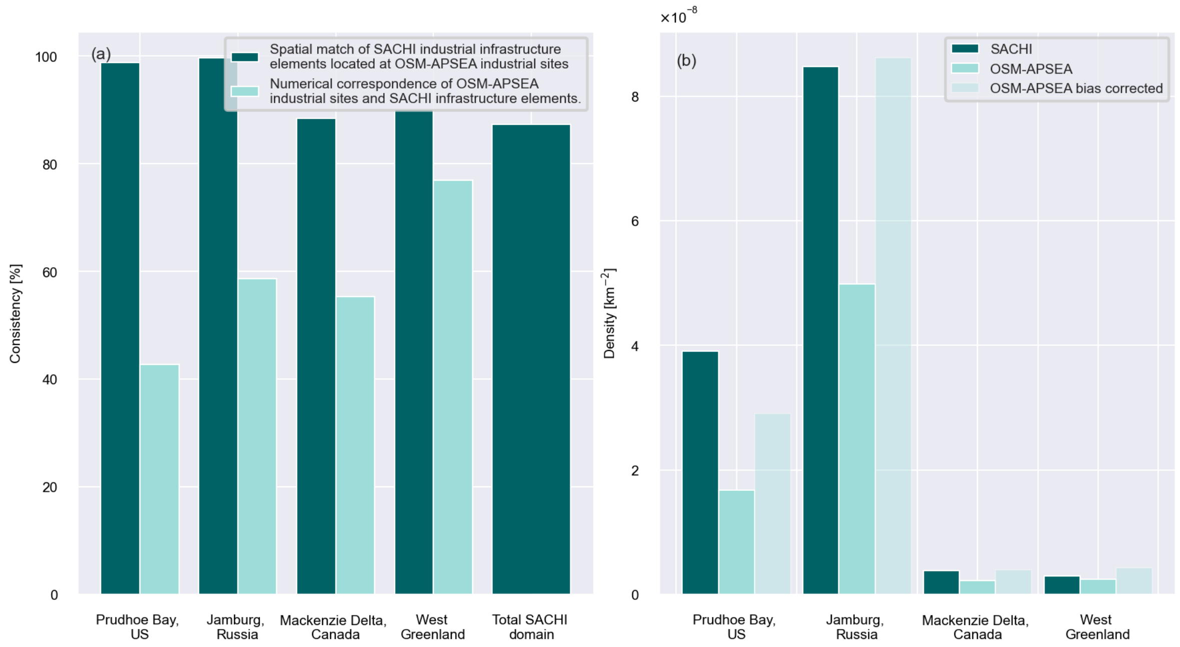

Supplementary Fig. 6: A comparison between the OSM-APSEA database and the Arctic coastal infrastructure satellite product (SACHI) reveals a match of more than 85% (true-positive) for selected test regions as well as for the total SACHI domain (a). This means that wherever OSM-APSEA indicates an industrial site, the SACHI database indicates in more than 85% of the cases the presence of industrial infrastructure. For the test regions, individual industrial infrastructure elements were manually classified within the SACHI dataset to facilitate a direct comparison between the number of industrial sites and the number of infrastructure elements (b). This comparison shows that the number of industrial sites within OSM-APSEA is on average 40 ± 20% lower than the number of industrial infrastructure elements in SACHI. For the test regions the bias might be correct by a simple multiplier.

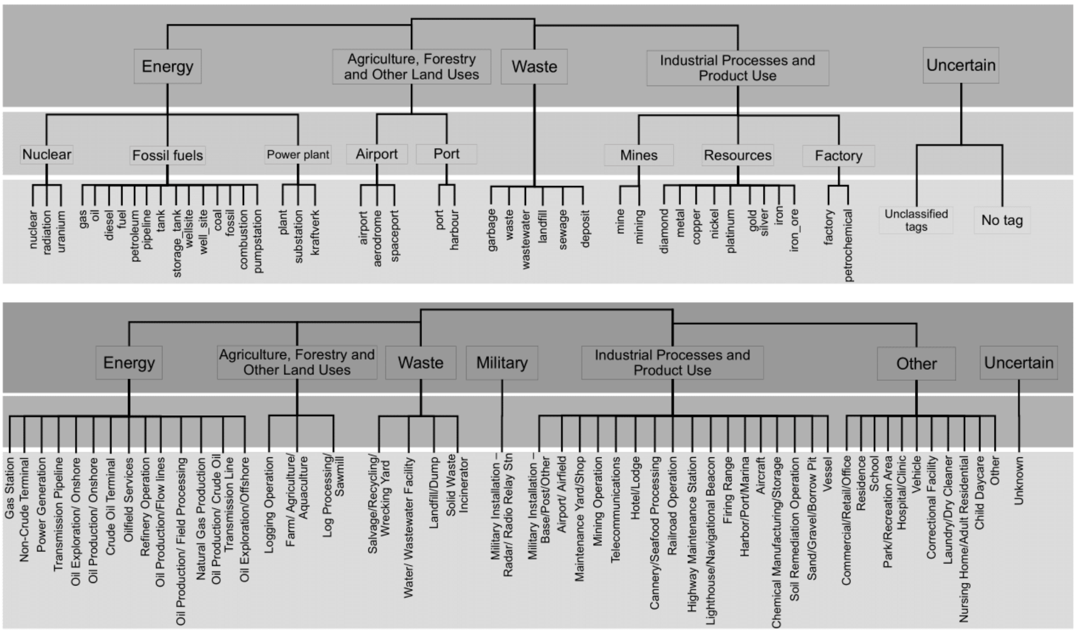

Supplementary Fig. 7: The dendrogramms illustrating the semantic classification performed on the database of industrial sites synergized from from OpenStreetMap (OSM) and the Nordregio Atlas of population, society and economy in the Arctic from 2019 (top) and the database of contaminated sites from the Contaminated Sites Program (CSP) in Alaska published by the U.S. Department of Environmental Conservation (bottom). After cleaning and translation of the site descriptions, the sites were classified into industrial sectors according to descriptions used by the Intergovernmental Panel on Climate Change (IPCC).

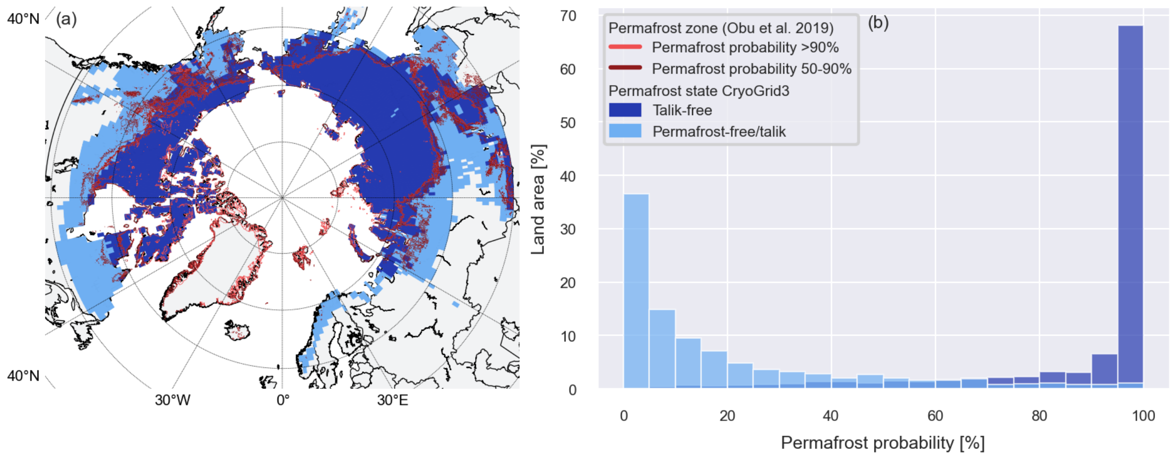

Supplementary Fig. 8: The map (a) shows the outline of the permafrost model domain delineated by permafrost occurrence probabilities (>50%) using the Northern Hemisphere Permafrost Map (NHPM) (32), underlain by persistent talik presence as simulated with the CryoGridLite model for the reference period (2000-2016). The histogram (b) shows the distributions of permafrost occurrence probabilities from the NHPM (32) for the CryoGridLite grid cells stimulated as talik-free and those with talik or permafrost-free between 2000 and 2016. Permafrost occurrence probabilities from the NHPM (32) were aggregated to the spatial resolution of the CryoGridLite grid cells (1 degree) by averaging. Map generated with Python using the Basemap Matplotlib library and the GSHHG datasets (33).

Supplementary References

1.) French, H. M. Terrain, land use and waste drilling fluid disposal problems, Arctic Canada. *Arctic* **33**(4), 794-806 (1980).

2.) Chuvilin, E. M., Yershov, E. M., Naletova, N. S. & Miklyaeva, E. S. The use of permafrost for the storage of oil and oil products. *Polar Record* **36**(198), 211-214 (2000).

3.) Abakumov, E. V. *et al.* Polycyclic aromatic hydrocarbons in insular and coastal soils of the Russian Arctic. *Eurasian Soil Science* **48**(12), 1300-1305 (2015).

4.) Thienpont, J. R. *et al.* Exploratory hydrocarbon drilling impacts to Arctic lake ecosystems. *PLoS One* **8**(11), e78875 (2013).

5.) Søndergaard, J., Elberling, B. & Asmund, G. Metal speciation and bioavailability in acid mine drainage from a high Arctic coal mine waste rock pile: Temporal variations assessed through high-resolution water sampling, geochemical modelling and DGT. *Cold regions science and technology* **54**(2), 89-96 (2008).

6.) Krivonogova, N. F. *et al.* Assessment of the state of hydraulic structures of the Bilibino Nuclear Power Plant. *Izvestiya Vserossiyskogo nauchno-issledovatelskogo instituta Gidrotekhniki im. Vedeneeva (in Russian),* **256**, 89-96 (2009).

7.) Johnson, D. Use of engineered landfills for arctic mine site reclamation. In *Proceedings of the Sixth International Conference on Mine Closure,* 495-502. Australian Centre for Geomechanics (2011).

8.) Artamonova, S. Y., Kozhevnikov, N. O. & Antonov, E. Y. Permafrost and groundwater settings at the site of “Kraton-3” peaceful underground nuclear explosion (Yakutia), from TEM data. *Russian Geology and Geophysics* **54**(5), 555-565 (2013).

9.) Ershov, E. D., Parmuzin, S. Y., Lobanov, S. Y. & Lopatin, V. V. “Problems of radioactive waste burial in perennially frozen ground” in *Proceedings of the 10th International Conference on Permafrost*, Phillips, M., Springman, S. M. & Arenson, L. U. Eds. (Swets and Zeitlinger, Lisse, Zurich, Switzerland, 2003) pp. 235-238.

10.) Biskaborn, B. K. *et al.* Effects of climate change and industrialization on Lake Bolshoe Toko, eastern Siberia. *Journal of Paleolimnology* **65***,* 1-18 (2021).

11.) Dowdall, M. *et al.* Assessment of the radiological impacts of historical coal mining operations on the environment of Ny-Ålesund, Svalbard. *Journal of environmental radioactivity* **71**(2), 101-114 (2004).

12.) Bach, L., Nørregaard, R. D., Hansen, V. & Gustavson, K. Review on environmental risk assessment of mining chemicals used for mineral separation in the mineral resources industry and recommendations for Greenland. *Scientific Report from DCE–Danish Centre for Environment and Energy*, 203 (2016).

13.) Edson, R., Johnson, G. L., Codispoti, L. A. & Curtin, T. ANWAP Science Team. The Arctic Nuclear Waste Assessment Program. *Oceanography* **10**(1), 4-10 (1997).

14.) Skorve, J. The environment of the nuclear test sites on Novaya Zemlya. *Science of the total environment* **202**(1-3), 167-172 (1997).

15.) Bond, M. J. & Carr, J. Permafrost thaw and implications for the fate and transport of tritium in the Canadian north. *Journal of environmental Radioactivity* **192**, 295-311 (2018).

16.) Kiselev, B. V., Khokholov, Yu. A. & Kaimonov, M. V. Erection of protective sarcophagus of semi-buried burial grounds for solid radioactive waste in the permafrost zone. *Geoekologiya. Inzhenernaya geologiya, gidrogeologiya, geokriologiya (in Russian)*, **3**, 255-260 (2010).

17.) Shirapova, S. D. On the prerequisites for creating threats to the natural environment and public health at the Khiagda uranium deposit. *Vestnik Buryatskogo Gosudarstvennogo Universiteta. Biologiya, Geografiya (in Russian)*, **4**, 83-85 (2010).

18.) Kachinskii, V. L., Zavgorodnyaya, Y. A. & Gennadiev, A. N. Hydrocarbon contamination of arctic tundra soils of the Bol'shoi Lyakhovskii Island (the Novosibirskie Islands). *Eurasian Soil Science,* **47**(2), 57-69 (2014).

19.) Straughn, R. O. The sanitary landfill in the Subarctic. *Arctic* **25**(1)*,* 40-48 (1972).

20.) Grebenets, V., Iurov, F. & Tolmanov, V. Negative changes in permafrost due to waste storage. In *EGU General Assembly Conference Abstracts,* 6824 (2020).

21.) Groisman, P. *et al.*, Northern Eurasia Future Initiative (NEFI): facing the challenges and pathways of global change in the twenty-first century. *Progress in Earth and Planetary Science* **4**(1), 1-48 (2017).

22.) McCarthy, K., Walker, L. & Vigoren, L. Subsurface fate of spilled petroleum hydrocarbons in continuous permafrost. *Cold regions science and technology* **38**(1), 43-54 (2004).

23.) Young, K. L. & Lund, K. An investigation of cadmium and lead from a High Arctic waste disposal site, Resolute Bay, Nunavut, Canada. *Hydrology Research* **37**(4-5), 441-453 (2006).

24.) Zenkevich, M., Prokofyev, V. & Yanovich, K. On the issue of the impact of oil pollution on the ecosystems of the Arctic zone of the Russian Federation. *Sistema znaniy: sovremennye modeli rasprostraneniya nauchnoy informatsii (in Russian)* (collection of scientific papers), 141-146 (2021).

25.) Revich, B. A. & Podolnaya, M. A. Thawing of permafrost may disturb historic cattle burial grounds in East Siberia. *Global health action* **4**(1), 8482 (2011).

26.) Filler, D. M., Kennicutt, M. C., Snape, I., Sweet, S. T. & Klein, A. G. “Arctic and Antarctic spills” in *Handbook of oil spill science and technology,* M. Fingas, Eds. (John Wiley & Sons, Inc. Hoboken 2015), pp. 495-512.

27.) Kim, L. ‘’Russian power plant spills thousands of tons of oil into Arctic region.’’ *NPR Environment, 2020.* Available online: https://www.npr.org/2020/06/04/869936256/russian-power-plant-spills-thousands-of-tons-of-oil-into-arctic-region?t=1591350190953 (accessed 17 July 2020*)*.

28.) Kramer, A. ‘’Russia confirms radioactive materials were involved in deadly blast.’’ *The New York Times,* 2019. Available online: https://nyti.ms/2ZU8aOo (accessed 17 July 2020*)*.

29.) Nilsen, T. The Independent Barents Observer, ‘’Arctic countries have been working together to step up nuclear accident preparedness.’’ *Arctic Today,* 2019*.* Available online: https://www.arctictoday.com/how-arctic-countries-have-begun-working-together-to-step-up-nuclear-accident-preparedness/ (accessed 17 July 2020).

30.) Tomskaya, L. A., Trofimova, L. N. & Kanaeva, A. N. Elimination of emergency spills of oil and oil products in permafrost. *Sistema tsennostey sovremennogo obshchestva (in Russian)*, **13** (2010).

31.) EIA, Crude oil production on the North Slope provided by the U.S. Energy Information Administration (2022), Available online: https://www.eia.gov/dnav/pet/hist/LeafHandler.ashx?n=pet&s=manfpak2&f=a (accessed March 2022).

32.) Obu, J. *et al.* Northern Hemisphere permafrost map based on TTOP modelling for 2000–2016 at 1 km2 scale. *Earth-Science Reviews,* **193**, 299-316 (2019).

33.) Wessel, P., & Smith, W. H. A global, self‐consistent, hierarchical, high‐resolution shoreline database. Journal of Geophysical Research: *Solid Earth*, **101**(B4), 8741-8743 (1996).
